# A connected community response to COVID-19 in Toronto

**DOI:** 10.1177/17579759211038258

**Published:** 2021-09-11

**Authors:** Garrett T. Morgan, Blake Poland, Suzanne F. Jackson, Anne Gloger, Sarah Luca, Norene Lach, Imara Ajani Rolston

**Affiliations:** 1 Department of Geography and Planning, University of Toronto, Toronto, Ontario, Canada; 2 Dalla Lana School of Public Health, University of Toronto, Toronto, Ontario, Canada; 3 Centre for Connected Communities, Toronto, Ontario, Canada

**Keywords:** COVID-19, community resilience, health promotion, connected community approach

## Abstract

In this commentary, we describe initial learnings from a community-based research project that explored how the relational space between residents and formal institutions in six marginalised communities in Toronto, Ontario, Canada impacted grassroots responses to the health and psycho-social stresses that were created and amplified by the coronavirus disease 2019 (COVID-19) pandemic. Our research found that grassroots community leaders stepped up to fill the gaps left by Toronto’s formal public health and emergency management systems and were essential for mitigating the psycho-social and socioeconomic impacts of the pandemic that exacerbated pre-existing inequities and systemic failures. We suggest that building community resilience in marginalised communities in Toronto can embody health promotion in action where community members, organisational, institutional and government players create the social infrastructure necessary to build on local assets and work together to promote health by strengthening community action, advocating for healthy public policy and creating supportive environments.

## Introduction

The coronavirus disease 2019 (COVID-19) pandemic offered an unprecedented natural experiment for studying community resilience in real time. Previous pandemics have demonstrated that while the mobilisation of biomedical interventions and health infrastructure are critical, the social infrastructure of community-led responses plays an equally crucial role in both flattening the curve of communicable illnesses and mitigating the psycho-social and socio-economic impact of a widespread pandemic (1). Formal public health and emergency response systems are not typically oriented to support community resilience building efforts before, during or after shock events, and may instead constrain grassroots capacity and action during moments of crisis. As a result, it often falls to communities themselves to use their own place-based infrastructure and social networks to address community issues that are all too often invisible to formal response systems.

In early summer 2020, the Dalla Lana School of Public Health at the University of Toronto and the Centre for Connected Communities (C3) undertook a community-based research project that explored community-led responses to the first wave of the COVID-19 pandemic in six marginalized communities in the city of Toronto through interviews with 46 grassroots leaders. What we heard in these conversations paints a mixed and high-stakes picture of exacerbated precarity and inequality, unprecedented efforts at mutual aid, and a sense that many municipal and formal organisations had abandoned communities. Issues of food security, the digital divide, precarious employment and challenges accessing critical information exacerbated already challenging circumstances for many grassroots leaders in each of the six target communities.

In this commentary, we argue that, consistent with health promotion, a connected community approach (CCA) can centre and amplify the voices, experiences, needs and assets of marginalised communities in their efforts to prepare for, respond to, recover from, regroup and bounce forward after any social, environmental and health crises.

## The role of resilient communities in crisis response

Despite calls for increased resilience, there remains little understanding of how community resilience is (a) effectively fostered in ways that are truly inclusive and address issues of marginalization (2); and (b) connected to and supported by formal response systems in ways that reinforce community efforts. Core to community resilience is an emphasis on the ways that grassroots leaders and residents draw upon existing strengths-based networks, relationships and supports to limit the impact of both the pandemic and measures taken to address it (1). Community resilience is a key feature of healthy, vibrant cities and, yet, most of the attention has focussed on resilience at the individual, organisational and social-ecological systems levels, especially during crises (3), rather than on collective community responses.

The task of building community resilience is often left to emergency preparedness, emergency response and ‘critical infrastructure’ professionals. The literature suggests this top-down approach is often ineffective (4) and antagonistic to the bottom-up community response that is common in the aftermath of shock events. Indeed, retrospective analyses of emergency response recovery in post-Katrina New Orleans (1), the aftermath of Hurricane Sandy (2) and extreme weather events in Appalachia (5) demonstrate the drawbacks of top-down resilience efforts, when not intentionally aligned with grassroots bottom-up responses. Beyond identifying the weaknesses of top-down approaches, these retrospective analyses highlight the need to bring greater attention to equity and the engagement of marginalized communities in resilience building efforts.

It is necessary to acknowledge that community resilience-building policies often use the language of community empowerment and engagement to download both the responsibility and accountability for the effective development and implementation of resilience strategies from state actors to community members themselves (6). The downloading of responsibilities is especially pernicious in the context of the current neoliberal political environment of fiscal constraints and austerity, which often undercut the very capacities and components of communities and individuals that have been shown to support resilience (7,8).

## A connected community approach

A CCA is a promising practice to be explored in relation to fostering community resilience. CCA is a set of interconnected principles and practices that support the authentic and meaningful connection of people who want to make a positive impact in their community. A CCA shares some affinities with asset-based community development, complexity theory, systems theory and collective impact (9). A CCA is an approach, not a rigid model, that frames community resilience as an emergent community development process. A CCA focuses on the interface between municipalities, institutions and grassroots community groups and develops the systems and processes that allow citizens and institutions to work together to effectively prepare for, respond to, recover from and bounce-forward after shock events.

The CCA emerged after two decades of community development work by the non-profit East Scarborough Storefront, operating in a marginalised inner suburban neighbourhood in Toronto (10). Storefront staff and partners experimented with a new approach to community development that sought to create a community social fabric that supports people, organisations, and initiatives to thrive (9). In 2012, based on the Storefront’s extensive impact on the community it served, staff began the process of articulating what made their approach unique and effective in East Scarborough and to explore ways in which their work could be applied in other communities with similar outcomes; resulting in the articulation of a CCA (9).

A CCA works on multiple levels simultaneously, both horizontally and vertically, to foster social connectedness to strengthen a community’s social fabric (9). It holds that ‘intentionally focussing on and strengthening the social connections and networks between and among organisations, these networks can be a catalyst to foment community-based social and economic development’ (9, p. 3). By supporting community building from the bottom up and inside out, the approach emphasises the central importance of community connectors or integrators (both individuals and grassroots organisations) that provide anchoring points for social network structures across levels and sectors (9). The idea is to weave together the community-building efforts of institutions and funders, grassroots groups and social service organisations, strengthening social capital, social fabric and, ultimately, the resilience of their community.

## CCA and health promotion

Intersectoral collaboration is a key health promotion strategy where ‘... intersectoral partnerships ... [are] crucial for community-engaged decision making and planning, creating health settings, galvanizing political commitment, resources and infrastructure...’ (11, p. 924). To achieve these kinds of intersectoral collaborations, a CCA focusses on the role of community-integrator organisations as coordinating entities between many different sectors and connecting municipalities, institutions and grassroots community members. In addition, community-integrators strengthen community action (another health promotion strategy) by developing the systems and processes that allow citizens and institutions to work together.

Another key connection between CCA and health promotion is the way that a CCA operates in a community as a setting, in a place-based and community-centred way. In *Settings for Health Promotion* (12), settings are identified as opportunities for the collaborative co-creation of healthier environments through changes in policy and practice that are undertaken in deep consultation and dialogue with workers, management and citizens/clients/users. CCA engages local citizens in dialogue with municipal and service provider stakeholders towards solving issues that face their local community and planning for the future.

CCA is health promotion in action, wherein community-focussed players build on their assets to work together and strengthen community action, develop and advocate for healthy public policies, and create supportive environments, aided (and in some cases coordinated) by the consistent presence of a ‘community integrator’ organisation (sometimes also referred to as a ‘community backbone organization’). These community integrator organisations convene spaces for dialogue and co-creation between residents and formal organisational/institutional structures in ways that put community assets, aspirations and voices more consistently at the centre of strategies, planning and action.

## CCA in COVID-19

Given the early evidence of the potential of CCAs for fostering community resilience in times of crisis, in July 2020 we launched a community-based research study entitled, Connected Communities in a Time of Physical Distancing (CCPD). We explored how grassroots work in six Toronto communities was helped or hindered by the formal social infrastructure that existed previously to the pandemic. We sought to understand what differences pre-existing social infrastructure, especially those characteristic of the CCA, make to communities’ capacities to prepare for, respond to, recover from and regroup after COVID-19, especially as communities interface with formal institutional responses. In particular, we were interested in unpacking: (a) What are the critical preconditions, such as equity and social cohesion, that affect a community’s crisis response? (b) What community building efforts were in place prior to the COVID-19 crisis that enabled communities to respond effectively? And (c) In what ways do formalised, top-down responses from municipalities, organisations and institutions, leverage/support or hinder community-based responses to COVID-19?

In this work, we paid particular attention to the responsiveness of local organisations, the city, emergency management, public health and other formal institutional systems through the eyes of grassroots community leaders. We learned that grassroots leaders in all six communities reacted quickly to respond to their neighbours’ needs for food, information and mental health support from the beginning of the pandemic. In some of the communities where the social infrastructure was already in place, grassroots efforts were more supported, connected and resourced (meeting more of the criteria for a CCA), there were more opportunities for a coordinated and collective response as well as support for the grassroots leaders. From the perspective of interviewees, the formalised, top-down responses to the pandemic from the City and service organisations faced many challenges trying to provide food and mental health support services to those in need in a timely way. Grassroots groups and leaders had to step in to fill the gaps.

The CCPD research project showed that, in six racialised low income neighbourhoods, grassroots groups were at the forefront of the pandemic response. This grassroots response helped reach people, provide information, support mental health, food and housing security outside of the formal emergency response strategy. Thus, by intentionally or unintentionally relying on grassroots responses, formal emergency response strategies exacerbated existing inequities and did not recognise the presence of community integrator organisations nor support a community engagement process. From a health promotion perspective, the emergency response systems did not integrate pre-existing collaborations between various service organisations and community organisations and leaders. Based on this work, we hope to advocate for recognition of and support for a CCA as a way to support communities to prepare for, respond to, recover from and bounce forward after major shock events and to integrate community responses in future City of Toronto emergency planning. Other work in progress spearheaded by our team describes in greater detail what a CCA is and how it fills a recognised gap in the disaster response literature that calls for better collaboration between civil society groups and formal institutional responses (Poland et al. unpublished) and the relational nature of this work (Jackson et al. unpublished).
